# The effects of the natural visual-aural attributes of urban green spaces on human behavior and emotional response

**DOI:** 10.3389/fpsyg.2023.1186806

**Published:** 2023-07-26

**Authors:** Yuting Yin, Yuhan Shao, Yu Meng, Yiying Hao

**Affiliations:** ^1^College of Architecture and Urban Planning, Tongji University, Shanghai, China; ^2^Acoustics and Vibration Group, Bureau Veritas, Manchester, United Kingdom

**Keywords:** nature-based solutions, visual and aural attributes, urban green spaces, behavior and emotional responses, perceptions

## Abstract

**Introduction:**

Nature-based solutions (NBS) have been used to address a wide range of urban environmental challenges, an important aspect of which is to improve human health and well-being. However, most relevant studies focus either on what positive influences nature may have or on identifying what natural factors can have these benefits. Few have investigated the sensory composition of nature and the effects of nature in different sensory aspects on human health. Setting out from the multi-sensory perspective, this study aims to explore human behavior and emotional response from visual and aural contact with urban nature.

**Methods:**

Taking Jiangjia Art Garden in Chengdu as an example, natural attributes such as its visual (landscape) and aural (sound source) characteristics as well as people’s activities (behavioral responses) were measured by on-site mapping analysis. This was done while a questionnaire-based survey was conducted to investigate people’s emotional responses regarding their overall satisfaction, pleasantness, calm, and agreeableness.

**Results:**

The results indicated that nature-dominated visual landscapes such as grassland, waterscapes, and woodlands, as well as natural sounds such as bird sounds, chirp sounds, and wind sounds were found to be positively correlated to the vitality of activities and people’s emotional status. Regarding behavioral responses, it was shown that grasslands and woodlands are more likely to be attractive places for recreation, and the vitality measured became extremely high when these two were paired with lakes. As for the emotional responses, people’s perceived overall satisfaction, calm, and agreeableness were equally reflected in their behavioral patterns, suggesting a strong relationship with natural factors.

**Discussion:**

The research findings were visually presented in behavior and emotional maps to provide direct cues of informing the future design of high-quality urban green spaces and promoting the application of aural-visual experience in the design of urban nature areas.

## 1. Introduction

“Nature-based solutions (NBS) are actions inspired by, supported by, or copied from nature” (Cecchi et al., 2015; Cohen-Shacham et al., 2019). The term has been used to address a wide range of urban environmental challenges, an important aspect of which is improving health and well-being. Nature has long been confirmed to have psychological benefits such as restoring depleted attentional resources (Yang D. et al., 2022; Yang Y. et al., 2022), mitigating mental stress (Johnsen, 2013), and arousing a positive affect (Barton and Pretty, 2010).

Sensory perceptions, behavioral patterns, and emotional responses of humans constitute their experiences in urban landscapes (Shao et al., 2022). However, to date, there is little evidence that describes the relationship of the three from the perspective of NBS. This study intends to explore the benefits of nature through the visual-aural aspects of urban green spaces, a typical nature-dominated urban space, on behavior and emotional response, so as to inform the design of urban green spaces in a way that benefits urban residents.

### 1.1. The benefits of nature on human health and well-being

Awareness regarding the various aspects of NBS has continuously developed throughout history, from healing gardens (see Ulrich and Parsons, 1992; Marcus and Barnes, 1999) to a broader concept of restorative environments (see Kaplan and Talbot, 1983; Ulrich, 1983; Kaplan and Kaplan, 1989), all the way to the more generalized “Healthy Cities Movements” (World Health Organisation, 1948). Nevertheless, it was not formalized until 1984, when the first report about the measurable effects of nature’s influence on health was published (Ulrich, 1984). Ulrich then further conceptualized his findings (Ulrich, 1979, 1981, 1983) into a psycho-evolutionary framework he called a stress reduction framework (SRF), stating that psychology undergoes positive changes based on emotional state, including reduced levels of negatively toned feelings such as fear or anger, and increases in positively toned feelings (Zuckerman, 1977; Ulrich, 1979; Ulrich et al., 1991). Another significant study of relevance that contributed greatly to restorative environment theory was the Outdoor Challenge Project, which was conducted (Kaplan and Talbot, 1983) with the purpose of finding convincing evidence that increased exposure to the wilderness offers considerable and lasting benefits for a variety of individuals. This program laid a foundation for Kaplan and Kaplan’s (Kaplan and Talbot, 1983; Kaplan and Kaplan, 1989; Kaplan, 1995; Altman and Wohlwill, 2012) attention restoration theory (ART) through proposing several factors relevant to people’s restorative experiences that are not unique to the wilderness setting (Kaplan and Talbot, 1983). These were later formalized into four major components in ART: being away, extent, fascination, and compatibility, all having restorative effects on attention (Kaplan and Talbot, 1983; Kaplan and Kaplan, 1989).

Besides exploring what benefits nature may induce, attention has also been paid to investigating what exact natural settings and factors can have or be designed to have the same benefits. For example, views of nature from a window at home or in the workplace (Ulrich et al., 1991; Kaplan, 1993; Tennessen and Cimprich, 1995; Kaplan, 2001) or just a glimpse of a small park on the way to work (Whyte, 1980) might have a positive influence on emotions and thus lead to an increased level of psychological health. Viewing nature from a window at home or in the workplace can support micro-restorative experiences (Ulrich et al., 1991; Kaplan, 1993; Tennessen and Cimprich, 1995; Kaplan, 2001). Ekkekakis et al. (2000) found that traveling through a natural setting for 10–15 min may provide a respite that, although brief, interrupts the process of resource depletion and promote a shift toward increased activation and positive moods (see also van den Berg et al., 2007; Tyrvainen et al., 2014). Grass, bushes, trees, and flower beds are highly predictive of psychological benefits (Nordh et al., 2009, 2013). It was found that ratings for restoration likelihood often increase with the number of street trees and the presence of flower beds (Lindal and Hartig, 2015). This is also the case for urban settings containing these natural factors, such as small-scale pocket parks within neighborhoods (Nordh et al., 2009, 2013), private gardens (Ivarsson and Hagerhall, 2008), and residential and commercial streets (Todorova et al., 2004; Lindal and Hartig, 2015; Yin et al., 2022).

### 1.2. Exploring the benefits of nature through human perceptions and behavior

Visual and aural senses are the most significant ways people interact with nature (Francomano et al., 2022). This also explains why most studies used photos (Thake et al., 2017), videos and recordings (Snell et al., 2019), simulated settings (Yu et al., 2017; Lafay et al., 2019), and real environments (Yang D. et al., 2022; Yang Y. et al., 2022) as stimuli to explore the benefits of nature on behavioral and emotional response. In soundscape research, many efforts have been made to explore the factors that influence people’s psychological health, the most of which are related to natural soundscape. Similar to visual landscape studies, existing research is mostly concerned either with identifying what natural settings or what natural sound sources are aurally beneficial to people. In general, the more urban the setting, the less natural the sounds it contains and the more negative the emotions it causes (Zhu et al., 2021). Protected areas, such as forest parks and heritage sites at the national level, as well as urban green spaces (Liu et al., 2019) including city parks (Sun et al., 2012), leisure green spaces (Zhang et al., 2022), and waterfront (Zhong et al., 2023) at the city level all contain rich and diverse natural sounds that are normally regarded as capable of bringing users positive aural landscape experiences. Though individual differences may exist, natural sounds in urban green spaces such as birdsong, cicadas, flowing water, and the sound of breeze blowing through leaves have positive benefits and contribute significantly to soothing stress (Zhong et al., 2023). In a study focusing on residents’ preferred sound sources in urban parks, researchers found that all types of residents had a high preference for natural sounds, especially the elder group (Gong et al., 2022).

Visual and aural landscapes also have impacts on behavior (Meng and Kang, 2016). Visual aspects affect behavior normally in indirect ways through the mediator “preference,” which is often defined as “liking” (Peschardt and Stigsdotter, 2013) or finding locations esthetically pleasing (Hartig and Staats, 2006). People prefer to go to places with certain attributes to carry out recreational activities (i.e., walking, jogging, and exercising) (Larson et al., 2014), social activities (i.e., meeting with friends, playing with children, and interacting with others) and leisure and self-reflection activities (Widoretno and Youngmin, 2015). Urban settings dominated by natural features, such as city parks, pocket parks, gardens, and landscape boulevards were often mentioned as favorite places. Engagement with the soundscape in urban areas is also affected by the activity done (Sarwono et al., 2022) and vice versa, though scant evidence has emerged in soundscape research clarifying its relationship with activity type. The sound pressure level is one of the most important factors affecting choice of activity when it exceeds a certain range (Nilsson et al., 2007). There are also studies focusing on the effects of anthropological sounds. The presence of music, especially classical music, in open public spaces can encourage people to linger, to converse, and to hold eating/drinking events (Lepore et al., 2016). Sarwono et al. (2022) also found that talking with friends and playing with children are positively associated with anthropological sounds (i.e., people talking, footsteps, and slow music), while relaxing and reading activities are more likely to occur when the environments are rich in natural sound (i.e., water, wind, birds chirping, and rain).

### 1.3. The aim of this study

Though contributions were made to investigate the interaction between visual and aural attributes of nature and the emotions and behaviors triggered by them, very few have explored their mutual relationships and influences through a common framework. What’s more, these relationships have not yet been utilized in predicting emotional and behavioral responses due to the difficulty of obtaining large-scale perception data. This may also impede the efficient delivery of design instructions for improving natural urban environments.

Setting out from the NBS perspective, this study aims at exploring behavior and emotional responses to visual and aural contact with urban nature. Taking the UGS, Jiangjia Art Garden in Chengdu Outer Ring Ecological Zone as an example, natural attributes of its visual (landscape) aspect were calculated with space syntax and Quantum Geographic Information System (QGIS). This was done while a soundwalk and questionnaire-based survey were conducted to investigate aural characteristics (sound source compositions) of the study site and emotional responses regarding their overall satisfaction, pleasantness, calm, and agreeableness. Onsite observation and mapping were also carried out to indicate activities within the site. The obtained information was used together to formulate a stochastic model, so that the interactive elements of nature in the visual and aural aspects on behavioral and emotional response could be disclosed. These models were then used to predict and illustrate behavior and emotional response within the whole site. Results were then visualized as emotional and activity maps to develop design implications for how Jiangjia Art Garden could be improved by providing people with more beneficial experiences of nature.

## 2. Materials and methods

### 2.1. Research site

**Figure 1:**
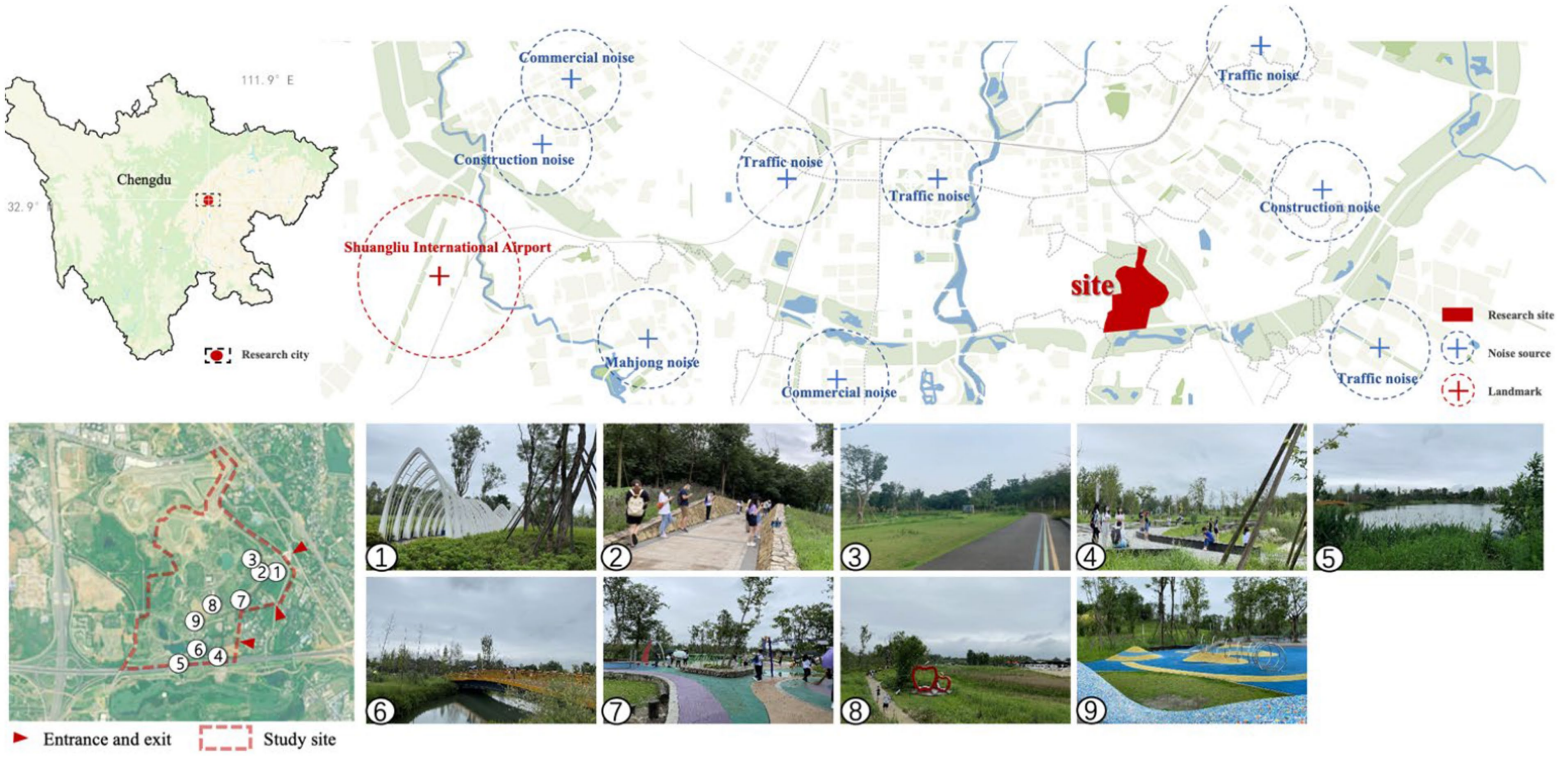
Research sites in Chengdu Outer Ring Ecological Zone: Jiangjia Art Garden.

This study selected Jiangjia Art Garden, a typical urban green space in Chengdu Outer Ring Ecological Zone, as a research site. Chengdu Outer Ring Ecological Zone is a circular urban natural area with over 100 square kilometers planned and constructed under the Park City Agenda promoted across China. As one of the benchmark demonstration projects, Jiangjia Art Garden is expected to become a leisure destination and a well-known landmark for the city of Chengdu. It is located in the south of the Chengdu Outer Ring Ecological Zone and is 1.32 square kilometers in size. Its visual landscape is dominated by lakes, woodland, herbals, and shrubs and is mostly covered by anthropological and natural sounds. Jiangjia Art Garden generally serves as a site for daily leisure activities, sightseeing, parent-child activities and exercising (Figure 1).

Nine measurement spots were selected in the Jiangjia Art Garden based on the following criteria: (1) they should be able to present the visual and acoustic features of the Jiangjia Art Garden; (2) they should cover sufficient distance for an over three-minute walk from the entrance to the first measurement spot to allow enough time for subjects to become mentally immersed in the setting (Aumond et al., 2017; Jo and Jeon, 2020). The three-minute walking distance should be the smallest interval between spots; and (3) there should be enough space for subjects to wander around within each spot. Thus, with the consideration of the size and the accessibility to the public, nine spots were chosen in Jiangjia Art Garden to measure its visual and aural landscape composition, as well as subjects’ emotional and behavioral responses to visual and aural environmental landscape stimuli. The scope of each spot was chosen as 50 m x 50 m to provide subjects with enough space to experience the surroundings without losing their sense of space (Figure 1).

### 2.2. Measurements

This study adopted a multi-method approach to investigate the visual and aural landscape compositions of Jiangjia Art Garden and their effects on behavior and emotional response. This included observation and mapping, calculations based on QGIS and space syntax, questionnaire-based onsite surveying, and focus group evaluation.

#### 2.2.1. Visual landscape compositions calculated with space syntax and QGIS

**Figure 2:**
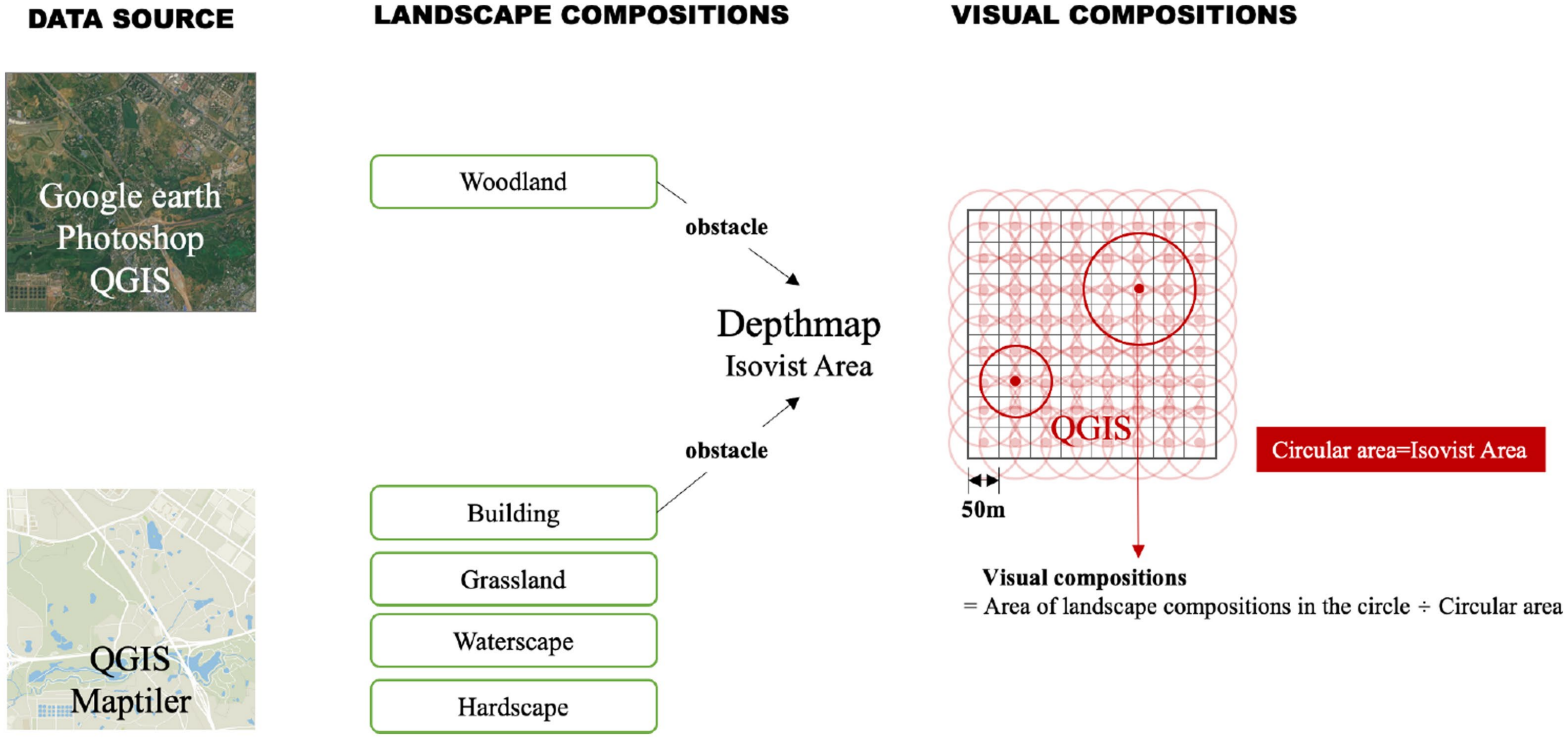
Measuring visual landscape calculations with space syntax and QGIS.

Given that the study was largely concerned with perception, visual landscape compositions were measured both in terms of the actual landscape distributions and what people can visually perceive within the site using QGIS and space syntax. The buildings and woodlands were set as visual obstacles, the boundaries of which were identified using QGIS Maptiler and Google satellite images to delimit the visible area. Space syntax software Depthmap was used to analyze the spatial visibility of the site over a 50 m × 50 m grid so that the visible area of each unit could be obtained. These were then imported into QGIS to generate the corresponding vector circles. The area of grasslands (G), waterscapes (Wa), hardscapes (H) and woodlands (Wo) within each circle were calculated to indicate what landscape compositions of Jiangjia Art Garden people visually perceived (Figure 2).

#### 2.2.2. Sound sources and emotions measured by soundwalk and questionnaire

Sound sources and emotional responses to the surrounding environment were measured by a sound walk and a questionnaire-based onsite survey conducted at each measurement spot.

A sound source is an attribute that can be characterized by identifying what sounds can be heard in the area and how dominant they are (ISO, 2018). Referring to international soundscape research standards (ISO, 2018), previous relevant studies (Shao et al., 2022) and the pilot study conducted at Jiajiang Art Garden, four types of sound sources (traffic sound, mechanical sound, anthropological sound, and natural sound) were provided as potential cues (Table 1). A sound walk is a standardized method widely used in soundscape research to measure aural perceptions (Liu et al., 2014). It was employed in this study to explore the sound sources perceived in each measurement spot of the site.

**Table 1:** Indicators of measuring perceived sound sources.

Indicators		Descriptions		
Sound sources	Traffic sound (S1)	Skateboards	Car horn	Rail
		Bicycle ring	Motor vehicles	Highway
		Heavy vehicles	Airplane	/
	Mechanical sound (S2)	Weeding	Construction	Alarm
		Radio	Loudspeaker	/
	Anthropologic sound (S3)	Exercising	Cleaning	Whistling
		Walking	Parent–child activities	Smiling
		Boating	Bells	Music
		Talking	Pedaling	Singing
	Natural sound (S4)	Insects	Tree murmur	Wind
		Croak	Twittering of birds	Ripple
		Fish dive	Barking dogs	Rain
Emotional responses	Pleasantness (E1)	The level of pleasure you feel in this environment		
	Agreeableness (E2)	How agreeable do you feel about this environment		
	Calm (E3)	The level of calmness you feel in this environment		
	The overall satisfaction (E4)	How satisfied do you feel about this environment		

One of the most classical theories in environmental psychology to understand human emotions is the Russell’s circumplex model (Russell, 1980). Two basic emotional dimensions, pleasantness (positive-negative) and arousal (high arousal-low arousal), were proposed in Russell’s model to, respectively, describe the trend and intensity of people’s emotional changes (Russell, 1980). The axis also includes a wide range of emotional indicators represent the different stages and levels of human emotional change. Among which, calm, annoying, pleasantness and excitement are regarded as the most typical four (Axelsson et al., 2010). Since excitement usually requires special stimuli (Beza, 2010), calm, annoying and pleasantness are thus adopted in the ISO (2018) and also in the perceived affective quality (PAQs, Axelsson et al., 2010) to measure human emotions in response to their perceived aural-visual qualities.

Consider semantic unity of and the difficulty of understanding the indicators, three emotional aspects (pleasantness, calm, and agreeableness) were selected and validated in a small-scale pilot study as efficient indicators of emotional response from visual and aural contact with the site. This was done while their overall satisfaction of Jiangjia Art Garden was obtained (Table 1).

The questionnaire consisted of two parts. The first part contained questions investigating the dominance of each sound source perceived at each spot (Zhang et al., 2018). The second part had four questions evaluating overall satisfaction, pleasantness, calm, and agreeableness of the site. Both parts used a five-level Likert scale (1–5 representing strongly disagree [1] to strongly agree [5]).

#### 2.2.3. Behavior vitality rated by the expert group through onsite observation

Onsite observation and mapping were also carried out by four trained surveyors to record activities at each measurement spot, represented as behavior vitality (V), used in this study to describe the diversity and intensity of activity. Behavior vitality was adopted from the concept of urban vitality which refers to the people and their activities throughout varied time schedules that can be observed in a specific space and is the product of the number and duration of various activities (Gehl, 1989). During the opening hours of Jiangjia Art Garden, surveyors ranked the behavior vitality of each spot at 2-h intervals from 8:00 to 17:00 (10:00, 12:00, 14:00 and 16:00) and also with a five-level Likert scale. The mean value of four surveyors’ ratings was then calculated as the results of behavior vitality for each spot.

### 2.3. Data collection

The sound walk and on-site questionnaire survey were conducted on sunny days with an Air Quality Index less than 60 and wind speed below 5 m/s during July 2021.

A total of 20 young adults were recruited to evaluate their perceived aural characteristics and their emotional affect at each measurement spot. Subjects were required to be between 20 and 30 years old, since previous studies suggested that age (Lyons, 1983) may influence emotional reactions under certain circumstances. This study is especially concerned with the aural-visual perceptions of young people since they are more sensitive to surrounding environments than other age groups (Kurakata et al., 2008). The recruited subjects all had normal or corrected-to-normal visual acuity, normal color vision, and hearing. The ratio of male to female subjects was controlled at 1:1. The survey was ethically approved, and informed consent was obtained from each subject in advance. Necessary training and introduction were also provided before the survey was formally taken.

The 20 recruited subjects were divided into four groups, and each group was led by one researcher to travel through the nine measurement spots in different orders to avoid having too many people appear at the same spot at the same time. Subjects were asked to take the sound walk and to wander around within each measurement spot by themselves to experience their surrounding environments for 5 min (Bahali and Tamer-Bayazit, 2017) and complete the questionnaire afterward. Each of the 20 subjects filled out the questionnaire nine times in each of the nine measurement spots; a total of 180 responses were collected. Noise data, such as an incomplete questionnaire or questionnaires with the same ratings for every indicator, were manually removed and the number of final valid responses was 139 (approximately 15 per spot). The sample size was in line with previous similar studies, which stated that the total number of valid responses should be over 100 (Kang and Zhang, 2010).

### 2.4. Data analysis and visualization

The analysis methods for exploring the relationship between human emotions and environmental aural-visual qualities normally conducted in two ways. The first one is to investigate the influencing factors of people’s positive or negative emotions through correlation analysis (Kong et al., 2022). However, it is only suitable for revealing the influence of a certain or a few of certain dimensions without presenting their interactions. Another way is to build a linear regression model to predict the overall environmental satisfaction or people’s emotional responses based on environmental aural-visual qualities. It can easily describe the complex relations existed between multi-dimensional environmental quality (Shao et al., 2022), but with an obvious disadvantage: the modeling method can be difficult to comprehend in design practices. Thus, this study decided to use the modeling method together with the mapping method, so that the relationship between environmental visual-aural qualities and human emotional and behavior responses can be directly presented.

Data analysis was conducted at two levels: the nine measurement spots and the case study site, while the visualization process was only taken at the site. The information regarding activity, sound source, and emotional response was collected at the spot level. This was then used together with visual characteristics of spots to formulate a stochastic model describing the relation between visual/aural landscape compositions and emotional reaction. Similarly, another mathematical model indicating the influences of visual and aural characteristics on behavioral reactions was also established. These models were then employed to generate the emotional and behavior map for the whole site using QGIS. Design implications to provide people with more appealing experiences can then be developed based on the mapping results describing behaviors and emotions in response to the visual and aural stimuli of Jiangjia Art Garden.

## 3. Results

### 3.1. Manipulation checks

Questionnaire data were analyzed using SPSS V26.0 and examined for internal consistency with Cronbach’s alpha (Bujang et al., 2018). Calculation of internal consistency (Cronbach’s α) was the preferred measure of inter-rater reliability when cases were rated in terms of an interval variable or interval-like variable, such as the Likert scale used in the questionnaire. The α values of the four emotional indicators and four sound-source indicators measured at the nine measurement spots suggested sufficient internal consistency (Cronbach’s α > 0.7), thereby guaranteeing the reliability of the obtained ratings.

### 3.2. Descriptive analysis

General descriptions of the measured visual-aural landscape characteristics and the obtained emotional and behavioral responses are shown in Table 2.

**Table 2:** Descriptive analysis of the obtained data.

		Wo	G	Wa	H	V	S1	S2	S3	S4	E1	E2	E3	E4
Spot 1	Mean	17.0%	58.0%	0.0%	25.0%	2.0	3.5	1.9	2.3	4.9	3.7	1.7	3.3	3.6
	Std. Dev.	–	–	–	–	–	0.7	0.8	0.9	0.3	0.5	0.6	1.0	0.5
Spot 2	Mean	23.0%	53.0%	0.0%	8.0%	4.0	2.5	2.8	4.1	3.9	3.3	1.6	2.3	3.4
	Std. Dev.	–	–	–	–	–	0.8	1.3	0.7	0.7	0.5	0.7	1.1	0.5
Spot 3	Mean	0.0%	33.0%	48.0%	13.0%	2.0	3.1	1.7	4.3	4.0	3.3	1.8	2.3	3.7
	Std. Dev.	–	–	–	–	–	0.9	0.9	0.7	0.8	0.7	0.8	0.9	0.5
Spot 4	Mean	0.0%	80.0%	4.0%	0.0%	1.0	4.1	2.9	3.2	3.7	2.8	2.7	2.3	3.3
	Std. Dev.	–	–	–	–	–	0.7	1.1	1.2	0.8	0.9	1.0	1.0	0.4
Spot 5	Mean	22.0%	57.0%	6.0%	0.0%	3.0	4.5	1.2	1.9	4.5	2.8	2.9	2.2	3.1
	Std. Dev.	–	–	–	–	–	0.6	0.4	0.9	0.5	0.5	0.6	1.4	0.3
Spot 6	Mean	6.0%	78.0%	7.0%	0.0%	0.0	4.6	1.2	2.6	4.3	2.4	2.7	1.9	3.0
	Std. Dev.	–	–	–	–	–	0.6	0.4	0.6	0.6	0.5	0.8	0.7	0.0
Spot 7	Mean	0.0%	75.0%	6.0%	8.0%	1.0	2.4	1.5	3.9	3.8	3.6	1.6	2.5	3.8
	Std. Dev.	–	–	–	–	–	0.9	0.5	1.1	0.6	0.7	0.7	1.1	0.4
Spot 8	Mean	14.0%	54.0%	16.0%	4.0%	1.0	3.8	1.3	2.6	4.8	3.8	2.0	3.0	3.4
	Std. Dev.	–	–	–	–	–	0.4	0.6	0.8	0.6	0.9	0.8	1.3	0.5
Spot 9	Mean	34.0%	52.0%	1.0%	0.0%	3.0	3.8	1.6	2.1	4.9	3.1	1.7	2.7	3.1
	Std. Dev.	–	–	–	–	–	0.4	0.8	0.7	0.3	0.5	0.8	0.8	0.3
Mean		–	–	–	–	1.9	3.6	1.8	3.0	4.3	3.2	2.1	2.5	3.4
The whole site		23.7%	51.7%	6.7%	17.7%	–	–	–	–	–	–	–	–	–

#### 3.2.1. Descriptive analysis of visual landscape compositions

**Figure 3:**
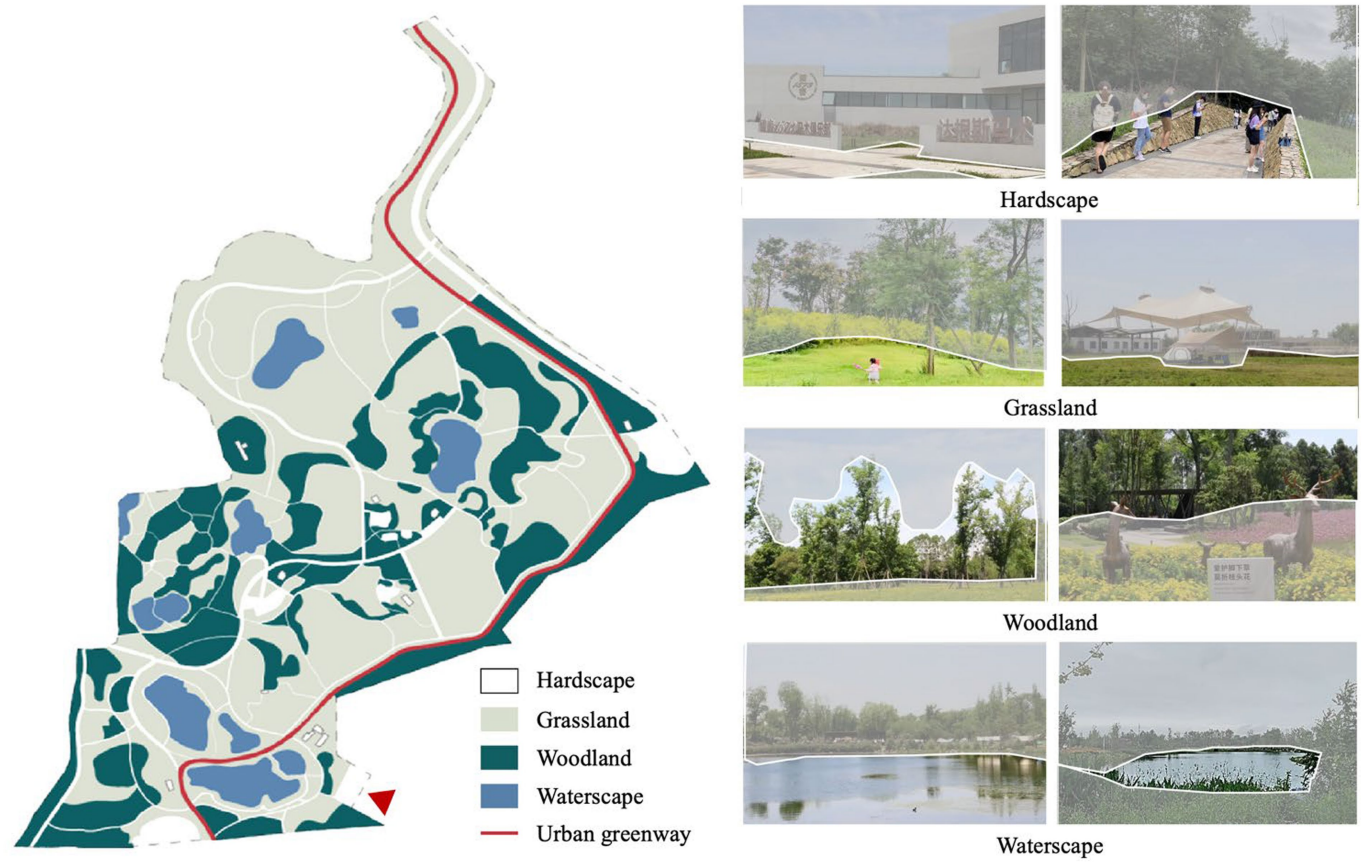
Visual landscape characteristics of Jiangjia Art Garden.

The actual landscape distribution of Jiangjia Art Garden indicates that the site is largely dominated by natural attributes such as grassland, woodland, and waterscape. Grasslands (51.9%) are the dominant feature, followed by woodlands (23.7%) and waterscape (6.7%), while hardscape such as pathways, squares, and playgrounds comprise around 17.7% of the total acreage. Grasslands are evenly distributed within the site, while woodlands are mainly centered along the main pathway and greenway. Waterscape is present mostly in the form of artificial lakes. The largest lake, which is composed of four pieces, was designed enclose to the main entrance of the site in a node-like fashion. One medium-sized and two small-scale lakes are located in the northeast and west of the site, respectively, and are set together with dense woodland. Another medium-sized lake is in the middle of a large-scale grassland at the northwest of the site. There are three nodes characterized by hardscape including one at the entrance functioning as a tourist transportation center, one in the middle that constitutes a landscape node surrounded by woodlands and lakes, and another one mainly composed of recreation facilities (Figure 3).

**Figure 4:**
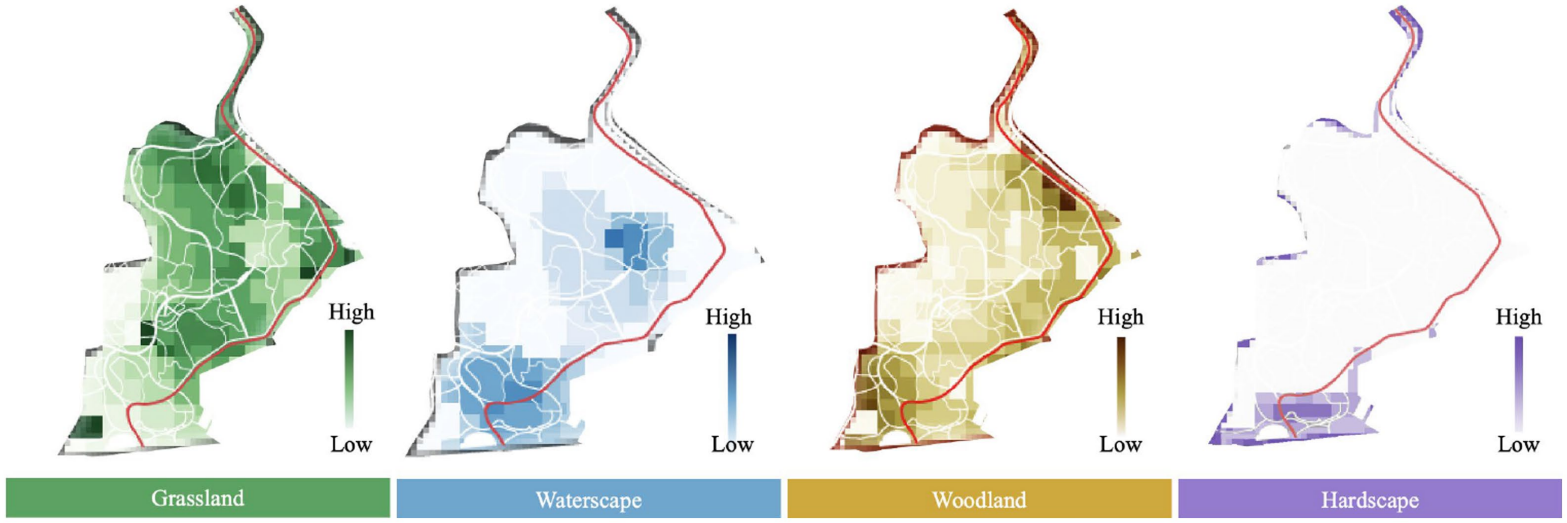
Visual landscape compositions of Jiangjia Art Garden.

In terms of visual landscape attributes perceived by people, grassland and woodland ranked at the top. People perceived the grasslands as centered at the southeast and the northwest of the site, which is mostly in line with their actual distribution. Also, the presence of waterscape was accurately perceived by the participants. However, differences were found between the actual distributions of woodland and hardscape and their perceived visual distribution. The aggregation of woodland in the western and northwestern area were not accurately perceived, while perceived woodland was mainly located close to the main entrance and the northeastern area. Similarly, hardscape was only perceived around the main entrance of the site, since it was not shown elsewhere within the site (Figure 4).

#### 3.2.2. Descriptive analysis of aural landscape compositions

**Figure 5:**
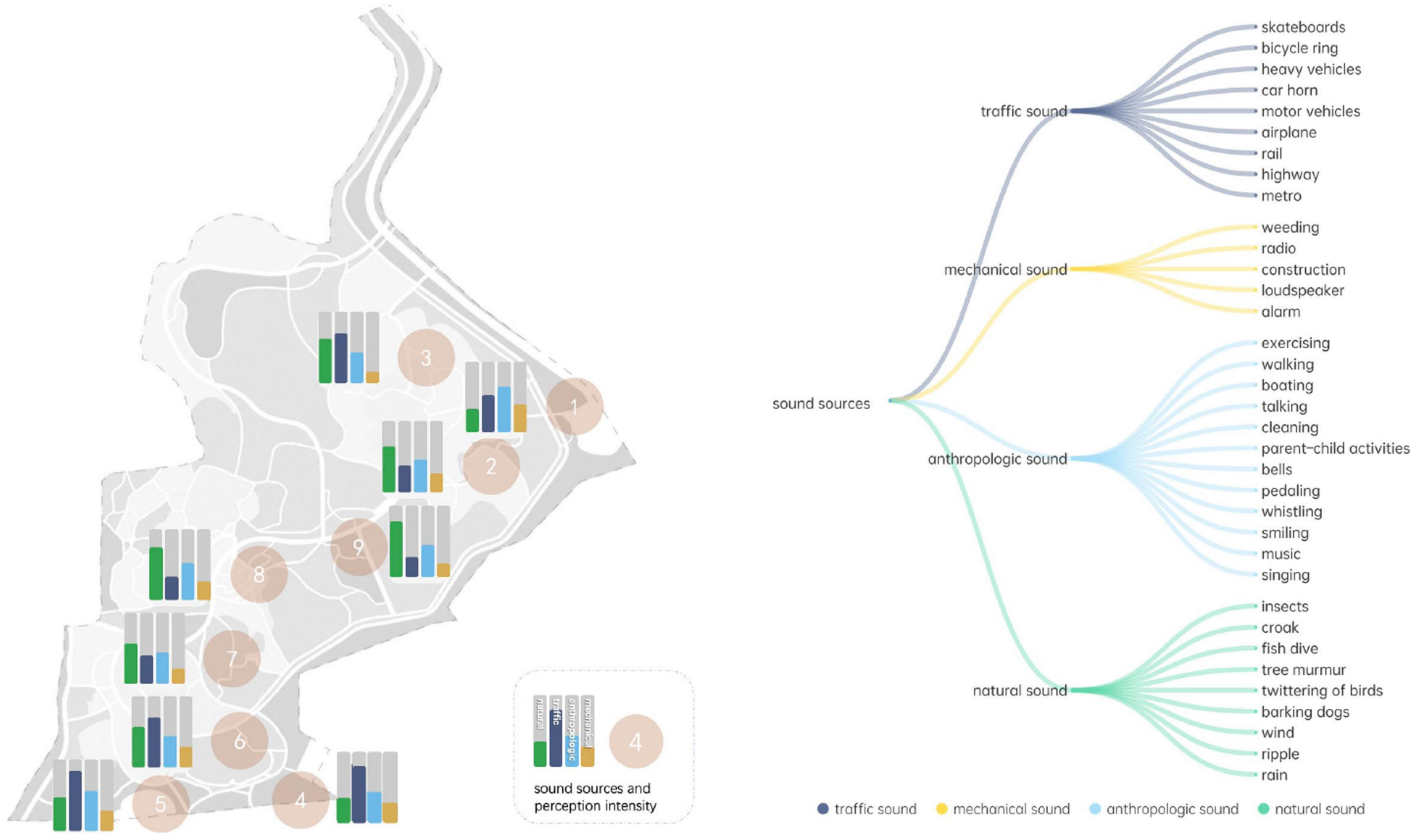
Aural landscape compositions of Jiangjia Art Garden.

Sound sources were rated by their dominancy at the nine measurement spots. Natural sounds were especially noticed at spot eight (4.8) and nine (4.9), locations that are relatively far from the main entrance and the surrounding railway. Natural sounds at spot four (3.7) were rated as the least obvious, since their locations are close to the outside of the site. At spots four (4.1), five (4.5), and six (4.6) near the main entrance where the highway passes through, the sound of traffic is considered most dominant. Mechanical sounds (avg. 1.8) were in general weak within the scope of Jiangjia Art Garden, with only spot two (2.8) and four (2.9) rated as higher, while anthropological sounds (avg. 3.0) such as walking, talking, and laughing were rated as evenly distributed across the nine measurement spots (Figure 5).

#### 3.2.3. Descriptive analysis of behavioral vitality

Based on the on-site observation, Jiangjia Art Garden can be divided into four areas with different functions and functional intensities. The area near the south entrance is dominated by social gathering functions. Open spaces and facilities are largely scattered in the middle eastern part of the site, an area that supports plenty of activities, while the middle western area mostly contains sightseeing and lake activities, such as boating and fishing. The northern part of the site, once farmland, has been transformed into an agriculture-themed entertainment space.

**Figure 6:**
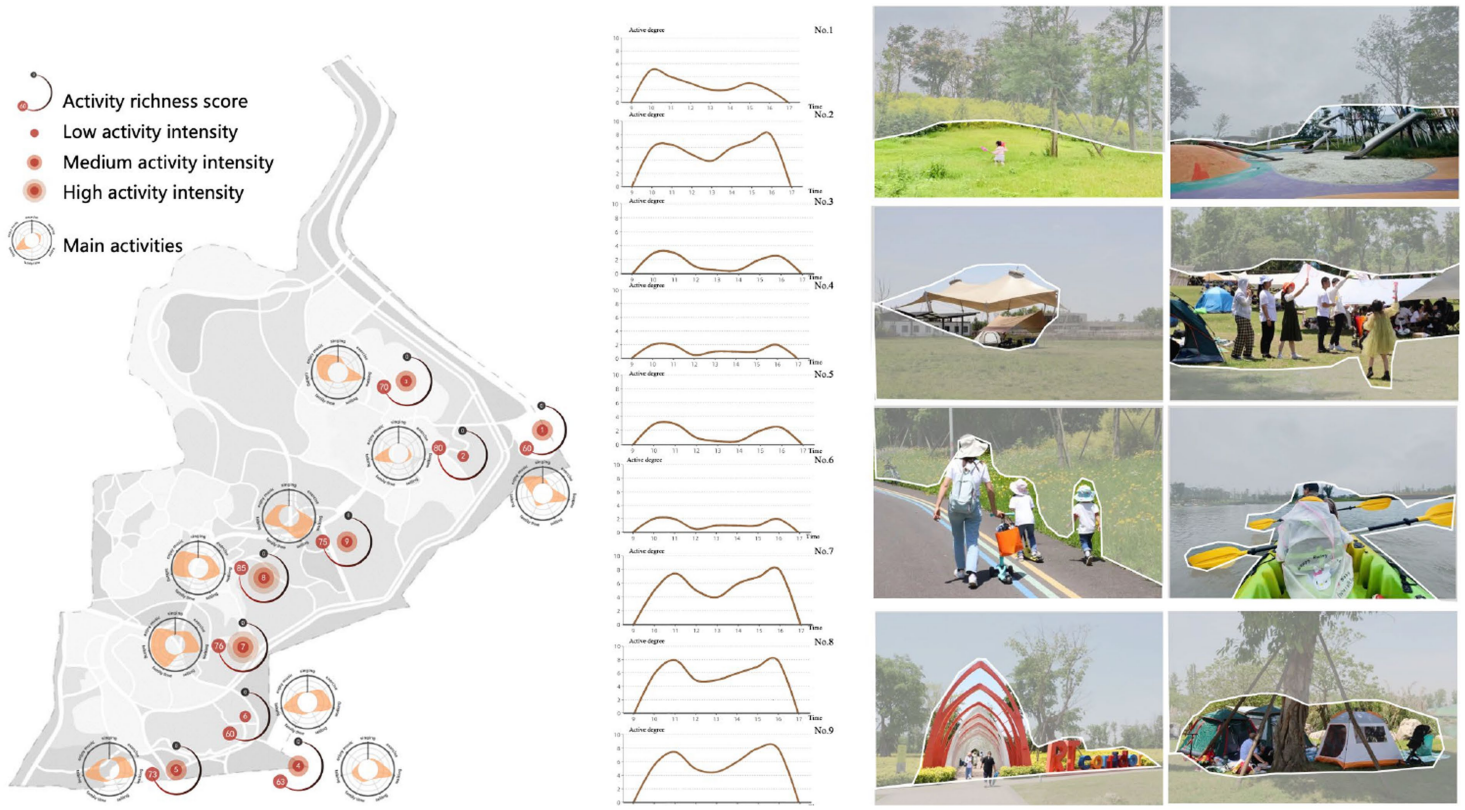
Human behavioral responses indicated by behavior vitality.

The results of behavior vitality measured by the diversity and intensity of activity occurring within each spot suggested that spot two (4.0) has the most diverse and intense activities including resting and talking with friends, photographing, dog walking, and children playing, especially from 8:30–10:00 and 15:00–16:00. Spots five (3.0) and nine (3.0), located at the southern and western side of the site, respectively, also were rated as having a medium level of behavior vitality. The former is dominated by exercising activities that peak between the hours of 8:30–9:30, while the latter is mainly composed of relaxing and resting activities between 8:30–10:00 and 15:00–16:00. Lower levels of vitality appeared at spots four (1.0), six (0.0) and eight (1.0), where mobile activities such as walking and sightseeing are prominent (Figure 6).

### 3.3. Human behavioral responses predicted by the visual-aural compositions

The measuring and rating results of visual and aural landscape characteristics were then analyzed through their behavior vitality to explore their influential factors and mechanisms.

Results suggested that behavior vitality in research sites is only affected by the visual components of nature with both woodland (*p* = 0.000 < 0.05) and grassland (*p* = 0.000 < 0.05) appearing to be related. The model describing their relation was established and its *R*^2^ was 0.613:


(1) V=3.002+0.058×Wo−0.029G

**Figure 7:**
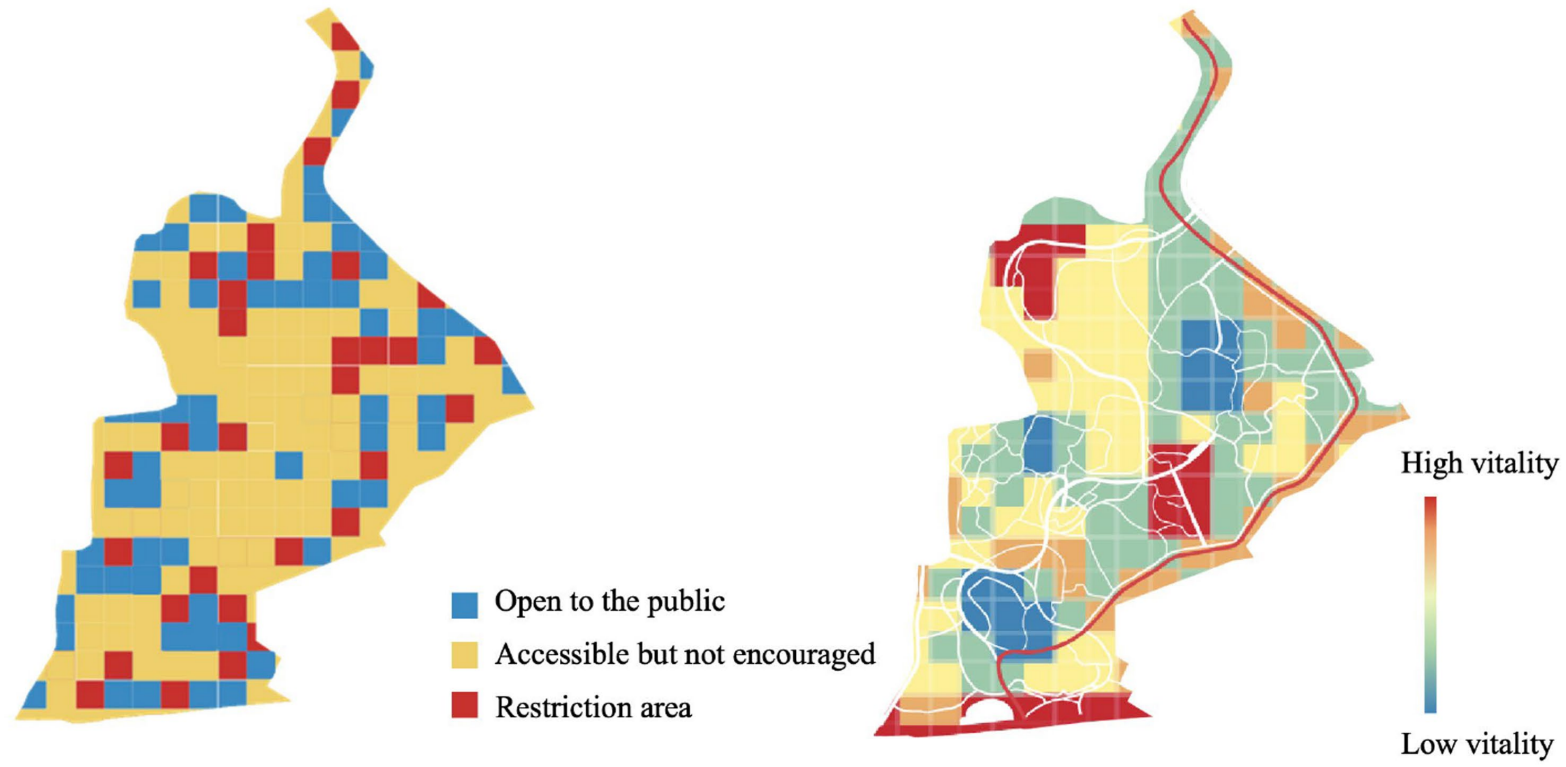
Accessible mapping (Left) and behavioral mapping (Right) for Jiangjia Art Garden.

The model was then used to predict behavior vitality of the whole site (Figure 7, Right) and compared with the accessible area of Jiajiang Art Garden (Figure 7, Left). The accessibility of the research site is classified into three levels: the open-to-the-public areas refer to those spaces mainly composed of pavements, greenways, and hardscapes; the accessible-but-not-encouraged areas include areas that are informally accessible to park users, such as grasslands, lakes, and woodlands; and the restricted areas are those that are physically inaccessible.

The comparison between two mapping results indicated that in general, activity can only partly be guided by the spatial design. The open-to-the-public areas do not usually attract the most intensive activity, while the highest vitality of activity was most observed in the accessible-but-not-encouraged areas, especially within grasslands and around waterscapes. The restricted areas, however, do not offer activities and thus present the lowest level of vitality.

### 3.4. Human emotional responses predicted by the visual-aural compositions

The natural components of visual landscapes, woodlands, and grasslands, as well as natural sounds, were confirmed to have significant influence on emotional response. Hardscape in the aspect of visual landscape and traffic sound in the aspect of aural landscape were also found to be related. The other three landscape compositions including waterscape, anthropologic, and mechanical sound, however, can hardly impacted the reported calm, agreeableness, pleasantness, and overall satisfaction (Table 3).

**Table 3:** Correlational analysis between the emotional responses and aural-visual indicators.

		S1	S2	S3	S4	Wo	Gr	Wa	H
E1	Correlation (Pearson’s r)	−0.436	0.031	0.238	0.220	0.049	−0.232	0.078	0.397
	Significant (p)	0.000	0.717	0.004	0.009	0.564	0.006	0.362	0.000
E2	Correlation (Pearson’s r)	0.481	−0.028	−0.164	0.008	−0.051	0.259	−0.129	−0.354
	Significant (p)	0.000	0.742	0.052	0.927	0.553	0.002	0.129	0.000
E3	Correlation (Pearson’s r)	−0.291	0.074	0.051	0.175	0.119	−0.120	−0.047	0.270
	Significant (p)	0.000	0.382	0.546	0.038	0.163	0.161	0.0.538	0.001
E4	Correlation (Pearson’s r)	−0.380	0.072	0.322	0.298	−0.183	−0.203	0.231	0.444
	Significant (p)	0.000	0.394	0.000	0.000	0.031	0.016	0.006	0.000

Results showed that two indicators describing the sound types, traffic sound and natural sound, as well as one visual indicator, hardscape, were strongly correlated with pleasantness. The model generated, with an *R*^2^ of 0.342, was as follows:


(2) E1=3.225−0.384×S1+0.273×S4+0.019×H

In terms of agreeableness, there was one visual and aural aspect that proved to be relevant, and a model with an *R*^2^ of 0.245 was found as:


(3) E2=0.922+0.371×S1−0.023×H

Only the aural components, traffic and natural sound, appeared to be active in the analysis of the relation between calm and the visual-aural components of the research sites. A model was constructed, and its *R*^2^ was 0.15:


(4) E3=2.113−0.378×S1+0.378×S4

The overall satisfaction was found to be influenced by the visual component of the woodlands and the aural components of traffic and natural sound. A linear model with an *R*^2^ of 0.434 appeared as:


(5) E4=3.513−0.01×Wo−0.266×S1+0.22×S4

The estimated coefficient values for all indicators and model-fitting information are listed in Table 4.

**Table 4:** Model fit and estimation of B coefficients of models.

	Model fit (*R*^2^)	Attribute	Estimate B	Standard error	*t*-value	*p*-value
E1	0.342	Constant	3.225	0.408	7.909	0.000
		S1	−0.385	0.066	−5.824	0.000
		S4	0.273	0.083	3.277	0.001
		H	0.019	0.009	2.110	0.037
E2	0.245	Constant	0.922	0.374	2.656	0.009
		S1	0.371	0.080	4.641	0.000
		H	−0.023	0.011	−2.027	0.045
E3	0.150	Constant	2.113	0.531	3.982	0.000
		S1	−0.378	0.086	−4.412	0.000
		S4	0.378	0.116	3.269	0.001
E4	0.434	Constant	3.513	0.199	17.609	0.000
		S1	−0.266	0.029	−9.129	0.000
		S4	0.220	0.044	4.948	0.000
		Wo	−0.10	0.003	−3.66	0.000

**Figure 8:**
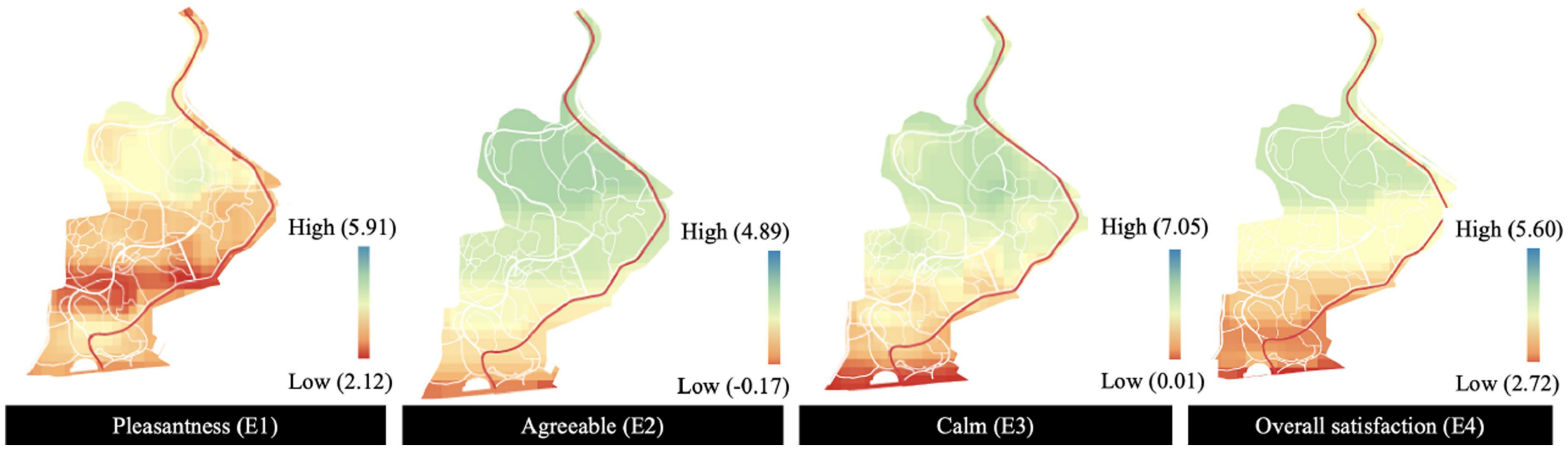
Emotional mapping for Jiangjia Art Garden.

This set of models was employed to visually represent the participant’s emotions (Figure 8). The tendencies of the four measured emotional aspects were consistent. In general, the northern area of the site was found to trigger better emotional responses than the southern area. The ratings of calm, agreeableness, and overall satisfaction decreased from north to south within the site, while the middle area, characterized by the waterscape, showed the highest level of pleasantness.

## 4. Discussion

### 4.1. Human behavioral responses and their component visual-aural landscape influences

This study used behavior vitality to describe the intensity and diversity of human activity in Jiangjia Art Garden. Also, it explored the relationship between activities and the visual-aural landscape attributes based on data collected at spot level, employing this relationship to construct a model to predict behavioral responses for the whole site.

In general, behavioral responses were only influenced by two visual natural components, woodlands and grasslands. Woodlands were found to encourage activity and grasslands can lessen the behavior vitality of the area, that are quite opposite with the findings in previous studies. Grasslands are generally regarded as preferred in UGSs for public activities because they provide open and wide spaces with soft “pavement” (Fu et al., 2019), while woodlands are normally less welcome because they provide limited spaces for gathering and staying activities. The survey was conducted in July, when the temperature is high, and the sunlight is strong. Compared to grasslands, woodlands can provide more shade and therefore are more attractive to visitors. Additionally, most grasslands in the site are posted with signs warning people not to step on the grasslands, which also may impede staying activities.

Considering both the distribution of visual landscape components and the accessibility and vitality mapping results, it can be concluded that activities do not usually comply with the designed functional requirements of spaces. People are most active where grasslands, water features, and woodlands coexist and where the accessible-but-not-encouraged spaces dominate. Results not only confirmed the accuracy of the generated model but also were in line with existing evidence suggesting that activities, especially informal ones, can largely be influenced by individual preference (Cheung, 2013; Cai et al., 2021; Xu et al., 2022). People continuously make minor modifications to the original arrangement of spaces, personalize their temporarily used spaces based on their needs and the rules of design, and thereby make these spaces their own (Thwaites et al., 2013). They prefer to choose their own activity areas, defining what is accessible for themselves, as shown in this study and in previous research (Kruize et al., 2019).

Another thing worth explaining is the vitality curves of spots 3 and 5, spots 4 and 6 appear to be similar with each other. This is possibly due to that the vitality average of each spot were processed through several calculations. During the opening hours of Jiangjia Art Garden, four surveyors observed and ranked the functional diversity and intensity of each spot at 2-h intervals from 8:00 to 17:00. The average of their ratings was used to present the diversity and intensity of each spot at each measurement time. The average of functional diversity and intensity was then calculated for each measurement spots to present their behavior vitality. Besides, the observation and evaluation were taken in three consecutive days in July 2021, and thus, the average of behavior vitality of three recorded days was calculated and used to generate the map. Though each spot may be rated as different in terms of diversity and intensity in each measurement day, they finally got the same average numbers at the 10:00, 12:00, 14:00, and 16:00 calculated within 3 days.

### 4.2. Human emotions and visual-aural landscape attributes

Emotional responses were also measured and investigated for the influence of various visual-aural landscape attributes. Models were then established, so as the relationship between pleasantness, calm, agreeableness, and the overall satisfaction subjects experienced in Jiangjia Art Garden and their relevant environmental components can be illustrated.

A total of four components, traffic and natural sounds in the aural aspect, as well as woodlands and hardscapes in the visual aspect, were identified as strongly related. Natural sounds showed consistent positive effects across the four emotional dimensions. The positive influences of natural sounds such as tree murmurs, twittering of birds, winds, and rain have been widely confirmed in previous research (Aletta et al., 2016; Ren et al., 2018; Buxton et al., 2021; Yang D. et al., 2022; Yang Y. et al., 2022). People’s perceptions are determined, or at least influenced, by their cognitions of former experiences in similar settings (Zajonc, 1980), combining their embedded feelings, images, and thoughts. Though urban parks are regarded as typical natural settings where users expect to experience nature, this kind of expectation can be relatively low for a park located in the central city of Chengdu which suffers from surrounding traffic and construction noises. However, this lower level of expectation may strengthen people’s positive responses since they take natural sounds perceived within the site as an extra surprise.

Another natural component of visual landscapes, woodlands, had a negative impact but only on overall satisfaction. While woodlands can provide shade in summer, they can also obstruct long-range view. Now that openness is one of the factors that affect environmental preference the most (Nasar and Jones, 1997) and the positive relation between preference and human emotions have been widely confirmed (Zhuang et al., 2021), these may provide supports for the inhibition influences of woodlands have on human emotions. Besides, the obstructed view in woodlands may also implies hidden danger and make people feel unsafe (Fu et al., 2019).

In terms of non-natural components, traffic sound and hardscapes both showed opposite impacts on emotion across the four models. Traffic sound had a negative influence on pleasantness, calm, and overall satisfaction, but enhanced agreeableness. This again supports the above assumptions about expectations regarding place identity (Yin et al., 2022). Hardscapes in visual landscape can promote pleasantness but can negatively influence agreeableness. Hardscapes normally occur in gathering spaces along with sculptures, fountains, and resting facilities. These can either provide people with objects of interest or opportunities to rest that both can contribute to a sense of pleasantness.

### 4.3. Limitations

Though the models and the mapping results in general meet with research purposes and no obvious contrast was found between outcomes of this study and previous research, there were limitations regarding the experimental design and data analysis processes. First, the survey was conducted in summer. The high outdoor temperature and the strong sunlight (Zhang et al., 2023) may have influenced behavior and emotions, especially decisions on where and when to conduct resting activities. These factors sometimes had an effect only on certain emotions, since four dimensions of emotional response were investigated. Under the same experimental conditions, participants’ standard responses may be slightly impacted but their comparison results should be equally as convincing. Although behavior, emotional response, and sound sources were all measured on a 5-point Likert scale, the four typical visual components of Jiangjia Art Garden were presented by a percentage. The differences on the components’ order of magnitudes may influence the correlation and modeling results. Other potential limitations may include the number of survey participants and the specific group (young adults) of participants. The final amount of analyzed data regarding behavior and emotional response was only slightly higher than the minimum required sample size due to the data-cleaning process. For studies using stochastic models, the larger the sample size, the higher the probability of obtaining a more accurate model. This may also lead to an unstable range of *R*^2^ in this study. Soundscape research normally rates an *R*^2^ above 0.3 (Yang, 2019; Lionello et al., 2020) as convincing for the linear model, but for many emotion studies, evidence suggests that models with an *R*^2^ over 0.1 can also provide sufficient reliability (Lionello et al., 2020). The overall results are therefore regarded as reliable, but models based on this study could further be improved by increasing the sample number in future attempts.

## 5. Conclusion

This study confirms that both seeing and hearing nature can have positive influences on behavioral and emotional response, thereby promoting the health and well-being of urban dwellers. Nature-dominated visual landscapes such as grasslands, lakes, and woods, as well as natural sounds such as bird sounds, chirp sounds, and wind sounds were found to be positively correlated with activity vitality and emotional status. Regarding behavioral responses, the evidence shows that grasslands and woodlands are more likely to attract activity and that vitality becomes extremely high when they are coupled with lakes. Though hard pavements provide spaces for people to gather and rest, they hardly promote activity vitality. In addition, places with more natural sounds have a higher volume of activity. Emotional response, overall satisfaction, calm, and agreeableness all show similar influences on behavioral patterns, suggesting a strong response to natural factors. The research findings could inform the future design of high-quality urban green spaces and promote the application of aural-visual experience in the design of urban nature areas.

## Data availability statement

The raw data supporting the conclusions of this article will be made available by the authors, without undue reservation.

## Ethics statement

The studies involving human participants were reviewed in accordance with the Declaration of Helsinki and approved by the Ethics Committee of Tongji University (protocol code 2020tjdx075 and date of approval 09/11/2020). The patients/participants provided their written informed consent to participate in this study.

## Author contributions

YY, YH, and YS: conceptualization. YY and YS: methodology and funding acquisition. YM: software, investigation, and visualization. YY and YH: validation, resources, data curation, writing—review and editing. YY, YM, and YS: formal analysis. YY and YM: writing—original draft preparation. YS: supervision and project administration. All authors contributed to the article and approved the submitted version.

## Funding

This research was funded by Shanghai Post-doctoral Excellence Program: 2021357; Restorative Urbanism Research Center (RURC), Joint Laboratory for International Cooperation on Eco-Urban Design, Tongji University (CAUP-UD-06); Shanghai Key Laboratory of Urban Design and Urban Science, NYU Shanghai Open Topic Grants (Grant No. 2022YTYin_LOUD) and Ministry of Science and Technology, High-end Foreign Experts Program (G2022133023L).

## Conflict of interest

YH was employed by the company Acoustics and Vibration Group, Bureau Veritas.

The remaining authors declare that the research was conducted in the absence of any commercial or financial relationships that could be construed as a potential conflict of interest.

## Publisher’s note

All claims expressed in this article are solely those of the authors and do not necessarily represent those of their affiliated organizations, or those of the publisher, the editors and the reviewers. Any product that may be evaluated in this article, or claim that may be made by its manufacturer, is not guaranteed or endorsed by the publisher.
